# Hippocampal calpain is required for the consolidation and reconsolidation but not extinction of contextual fear memory

**DOI:** 10.1186/s13041-017-0341-8

**Published:** 2017-12-19

**Authors:** Taikai Nagayoshi, Kiichiro Isoda, Nori Mamiya, Satoshi Kida

**Affiliations:** 1 Department of Bioscience, Faculty of Applied Bioscience, Tokyo University of Agriculture, Tokyo, Japan; 2 Core Research for Evolutional Science and Technology, Japan Science and Technology Agency, Saitama, Japan

**Keywords:** Calpain, Hippocampus, Fear conditioning, ALLN, c-fos

## Abstract

Memory consolidation, reconsolidation, and extinction have been shown to share similar molecular signatures, including new gene expression. Calpain is a Ca^2+^-dependent protease that exerts its effects through the proteolytic cleavage of target proteins. Neuron-specific conditional deletions of calpain 1 and 2 impair long-term potentiation in the hippocampus and spatial learning. Moreover, recent studies have suggested distinct roles of calpain 1 and 2 in synaptic plasticity. However, the role of hippocampal calpain in memory processes, especially memory consolidation, reconsolidation, and extinction, is still unclear. In the current study, we demonstrated the critical roles of hippocampal calpain in the consolidation, reconsolidation, and extinction of contextual fear memory in mice. We examined the effects of pharmacological inhibition of calpain in the hippocampus on these memory processes, using the N-Acetyl-Leu-Leu-norleucinal (ALLN; calpain 1 and 2 inhibitor). Microinfusion of ALLN into the dorsal hippocampus impaired long-term memory (24 h memory) without affecting short-term memory (2 h memory). Similarly, this pharmacological blockade of calpain in the dorsal hippocampus also disrupted reactivated memory but did not affect memory extinction. Importantly, the systemic administration of ALLN inhibited the induction of c-fos in the hippocampus, which is observed when memory is consolidated. Our observations showed that hippocampal calpain is required for the consolidation and reconsolidation of contextual fear memory. Further, the results suggested that calpain contributes to the regulation of new gene expression that is necessary for these memory processes as a regulator of Ca^2+^-signal transduction pathway.

## Introduction

Short-term memory (STM) is labile. The generation of stable long-term memory (LTM) requires the stabilization of a memory via a process known as memory consolidation [1–3]. The consolidated memory returns to the labile state following the retrieval and is re-stabilized through reconsolidation, which is a similar process to consolidation [4–7]. Conversely, the continuous or repeated retrieval of a conditioned fear memory initiates memory extinction, inhibiting fear responses [8–11]. The most common and critical biochemical signature of consolidation, reconsolidation, and extinction is the requirement for new gene expression [2, 7, 12–15].

Previous studies showed that protein degradation is involved in the molecular processes necessary for synaptic plasticity and learning and memory [16–20]. Calpain is a Ca^2+^-dependent cysteine protease involved in Ca^2+^ signaling pathway [21, 22]. It specifically cleaves substrates in neurons, including synaptic proteins such as membrane receptors, cytoskeletal proteins, postsynaptic density proproteins, and intracellular mediators, which are critical for synaptic function, and learning and memory [23–31]. Therefore, calpains have been known to contribute to neuronal processes, such as excitability, neurotransmitter release, synaptic plasticity, signal transduction, vesicular trafficking, structural stabilization, and gene transcription [32–34]. For instance, calpain specifically cleaves NMDA receptor 2B subunits (GluN2B), and p35, the neuronal-specific activator of cyclin-dependent kinase 5 (Cdk5) [25, 32, 35, 36], both of which play critical roles in learning and memory [37–40]. Calpain proteolysis targets the C-terminal of GluN2B, potentially changing the level of NMDA receptors and its activity at synapses [26]. Activated calpain cleaves the Cdk5 activator p35 in the N-terminal domains [41], generating a C-terminal-truncated product, i.e., p25, which plays critical roles in hippocampus-dependent memory [42, 43]. Importantly, neuron-specific conditional deletions of calpain 1 and 2 reduces dendritic branching complexity and spine density of hippocampal CA1 pyramidal neurons, which in turn impairs long-term potentiation (LTP) in the hippocampus and spatial learning [44]. Moreover, recent studies suggested that calpain 1 and 2 play distinct roles in synaptic plasticity [45]. However, the role of hippocampal calpain in memory processes, such as memory encoding, consolidation, reconsolidation, and extinction, remains unclear.

A contextual fear memory is an associative memory of a context with conditioned fear arising from a stimulus or event, such as an electric footshock. Memory consolidation and reconsolidation, but not extinction, of contextual fear requires the activation of gene expression in the hippocampus [13, 46–49]. In the present study, we clarified the role of hippocampal calpain in memory processes of contextual fear in mice. We analyzed the effects of the pharmacological inhibition of hippocampal calpain on memory consolidation, reconsolidation, and extinction of contextual fear. Further, since previous studies have suggested sex differences in molecular processes of learning and memory [50, 51], we also separately compared the role of calpains in female and male mice.

## Results

### Hippocampal calpain is required for the consolidation of contextual fear memory

**Fig. 1:**
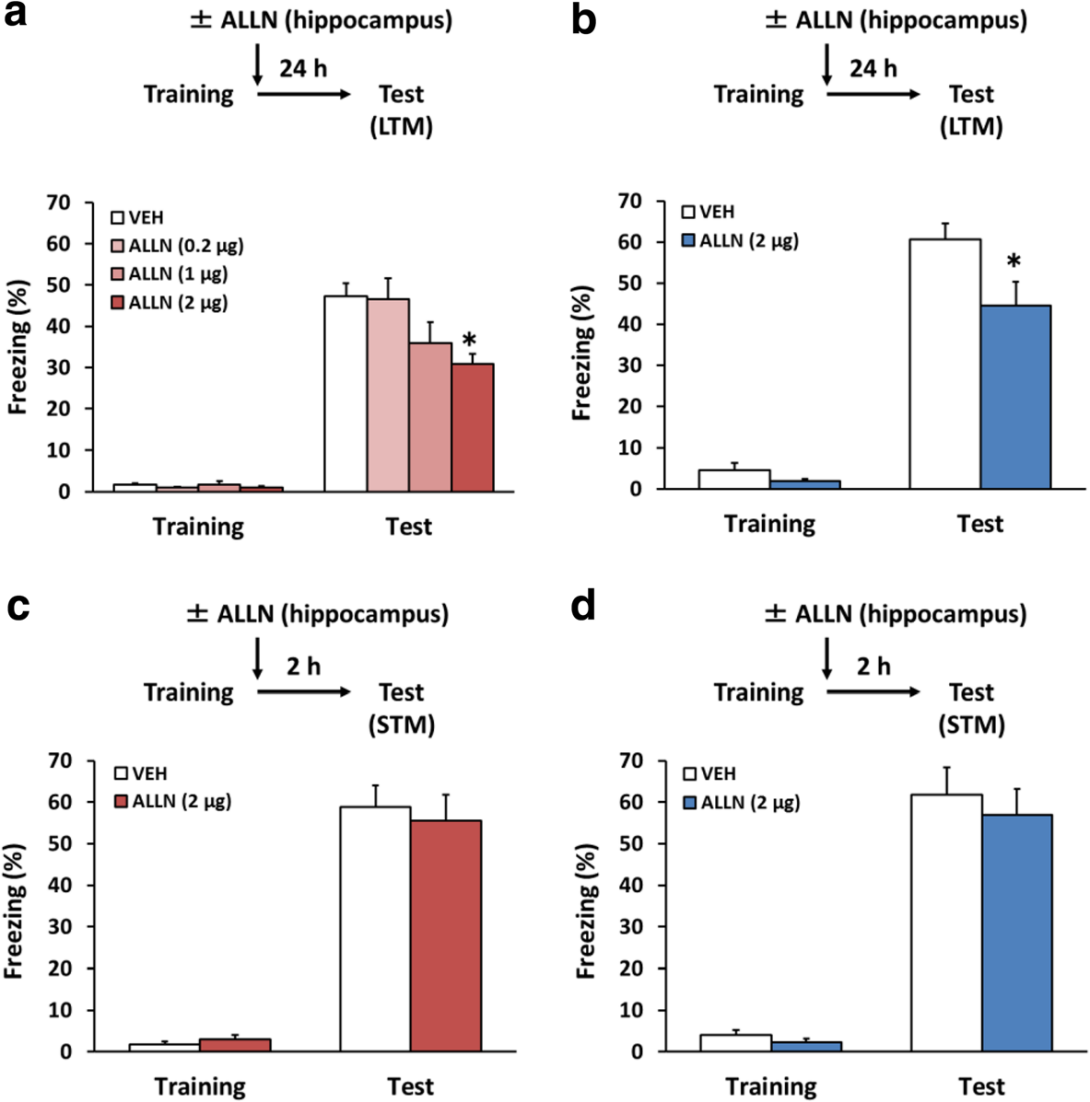
Inhibition of hippocampal calpain blocks the consolidation of contextual fear memory. **a** and **b** Effects of a microinfusion of a low-, middle-, or high-dose of N-Acetyl-Leu-Leu-norleucinal (ALLN) into the dorsal hippocampus immediately after the training on LTM in female (**a**) or male (**b**) mice (**a**: VEH, *n* = 28; ALLN 0.2 μg, *n* = 14; ALLN 1 μg, *n* = 10; ALLN 2 μg, *n* = 25; **b**: VEH, *n* = 14; ALLN, *n* = 11). **c** and **d** Effects of a microinfusion of ALLN into the dorsal hippocampus immediately after the training on STM in female (**c**) or male (**d**) mice (c: VEH, *n* = 11; ALLN, *n* = 10; d: VEH, *n* = 10; ALLN, *n* = 10). *p < 0.05, compared with the VEH group at the test. Error bars indicate standerd error of mean (SEM)

The hippocampus plays a crucial role in contextual fear conditioning and consolidation of this memory [46, 52–54]. To understand the role of calpain in memory formation, we investigated whether hippocampal calpain was required for the LTM of contextual fear. Importantly, the effects of a calpain inhibitor was separately examined in male and female mice, since recent studies suggested that sex differences are critical modulators of memory performance [50, 51]. The female mice were trained with a single footshock and tested 24 h later. They received a microinfusion of the calpain 1 and 2 inhibitor N-Acetyl-Leu-Leu-norleucinal (ALLN; low-dose, 0.2 μg/side; middle-dose, 1 μg/side; high-dose, 2 μg/side), or vehicle (VEH) into the dorsal hippocampus immediately after the training. A one-way analysis of variance (ANOVA) revealed a significant effect of drug (*F*(3,73) = 5.931, *p* < 0.05; Fig. 1a). Post hoc Newman-Keuls analysis revealed that mice treated with ALLN froze significantly less than VEH-treated mice in a dose-dependent manner (low-dose, *p* > 0.05; middle-dose, *p* > 0.05; high-dose, *p* < 0.05; Fig. 1a). Similarly, male mice treated with ALLN showed significantly less freezing compared to VEH-treated mice (one-way ANOVA, *F*(1,23) = 5.731, *p* < 0.05; Post hoc Newman-Keuls, *p* < 0.05; Fig. 1b). These observations indicated that the microinfusion of ALLN into the dorsal hippocampus impaired LTM of contextual fear.

Next, we examined the effect of an ALLN microinfusion on STM (2 h memory). The experiment was similar to that outlined in Fig. 1a and b, except that the mice were tested at 2 h after the training. A one-way ANOVA revealed no significant effect of drug (female, *F*(1,19) = 0.019, *p* > 0.05; male, *F*(1,18) = 0.287, *p* > 0.05; Fig. 1c and d). This observation indicated that female and male mice treated with ALLN showed normal STM. Taken together, these results demonstrated that the inhibition of hippocampal calpain by ALLN infusion impaired LTM formation of contextual fear, without affecting STM. In addition, the effects of sex differences of memory performance were not observed. Our observations suggested that hippocampal calpain is required for the consolidation of contextual fear memory.

### Hippocampal calpain is required for the reconsolidation of contextual fear memory

**Fig. 2:**
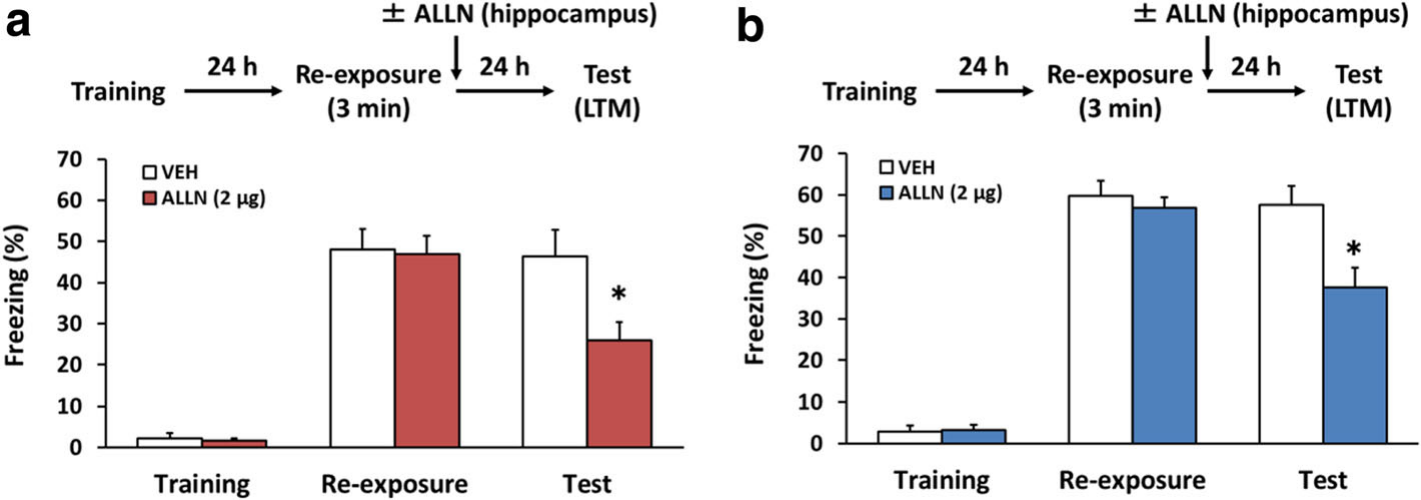
Inhibition of hippocampal calpain impairs the reconsolidation of contextual fear memory. Effects of a microinfusion of ALLN into the dorsal hippocampus immediately after the 3-min re-exposure on reactivated memory in female (**a**) or male (**b**) mice (**a**: VEH, *n* = 10; ALLN, *n* = 15; **b**: VEH, *n* = 10; ALLN, *n* = 12). *p < 0.05, compared with the VEH group at the test. Error bars indicate SEM

Reconsolidation involves similar molecular processes to consolidation [4–7, 13, 48]. Importantly, similarly to consolidation, reconsolidation of contextual fear memory depends on new gene expression in the hippocampus [13, 48, 55, 56]. Therefore, it is possible that hippocampal calpain is required for the reconsolidation of contextual fear memory. Next, we examined whether inhibition of hippocampal calpain affected the reconsolidation of contextual fear. Mice were trained, and re-exposed to the training context for 3 min (re-exposure) 24 h later. Reactivated fear memory was tested at 24 h after re-exposure (test). As illustrated in Fig. 1, the mice received a microinfusion of ALLN (2 μg/side) or VEH into the dorsal hippocampus immediately after the re-exposure. A two-way ANOVA revealed significant effects of drug (VEH vs. ALLN; female, *F*(1,46) = 7.201, *p* < 0.05; male, *F*(1,40) = 8.179, *p* < 0.05) and time (re-exposure vs. test; female, *F*(1,46) = 4.796, *p* < 0.05; male, *F*(1,40) = 7.139, *p* < 0.05), and a drug × time interaction (female, *F*(1,46) = 6.064, *p* < 0.05; male, *F*(1,40) = 4.39, *p* < 0.05; Fig. 2a and b). Post hoc Newman-Keuls analysis revealed that, during the test, ALLN-treated female and male mice froze significantly less than VEH-treated female and male mice, respectively (female, *p* < 0.05; male, *p* < 0.05; Fig. 2a and b). These results indicated that the inhibition of hippocampal calpain disrupted the reactivated contextual fear memory, which suggested that hippocampal calpain is required for the reconsolidation of contextual fear memory.

### Hippocampal calpain is not required for the extinction of contextual fear memory

**Fig. 3:**
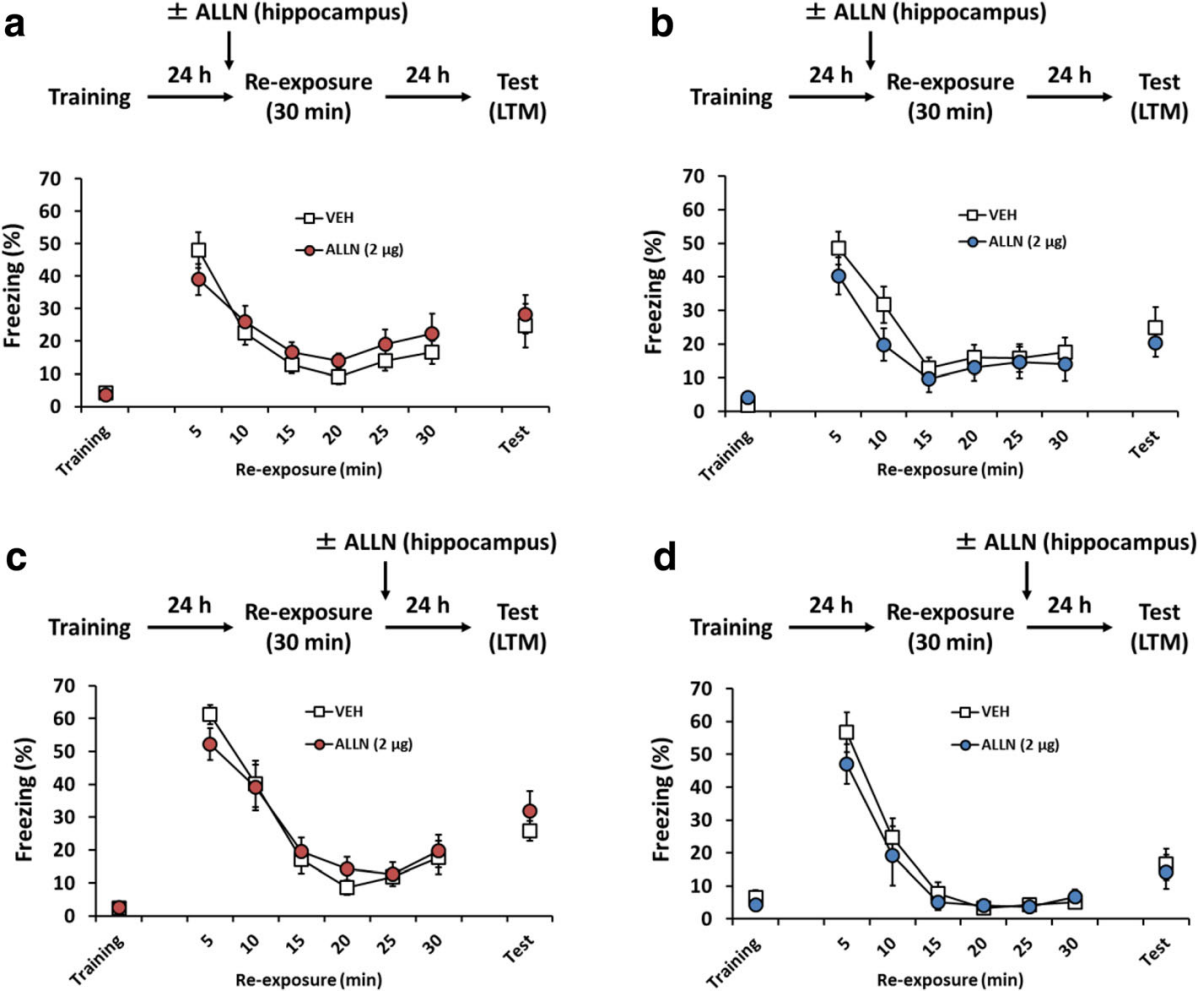
Inhibition of hippocampal calpain does not affect the long-term extinction of contextual fear memory. Effects of a microinfusion of ALLN into the dorsal hippocampus at 10 min before (**a** and **b**) or immediately after (**c** and **d**) the 30-min re-exposure on long-term extinction in female (**a** and **c**) or male (**b** and **d**) mice (**a**: VEH, *n* = 13; ALLN, *n* = 13; **b**: VEH, *n* = 10; ALLN, *n* = 11; **c**: VEH, *n* = 13; ALLN, *n* = 15; **d**: VEH, *n* = 10; ALLN, *n* = 11). Error bars indicate SEM

Since the long-term extinction of contextual fear memory requires new gene expression, it shows similar molecular signatures as consolidation and reconsolidation [15, 48, 57]. However, a previous study showed that the extinction of contextual fear memory requires gene expression in the amygdala and mPFC, but not the hippocampus [48], suggesting that the hippocampus shows distinct impacts on consolidation/reconsolidation and extinction. Therefore, we attempted to further clarify the role of hippocampal calpain in the extinction of contextual fear memory. The mice were trained, and 24 h later were re-exposed to the training context for 30 min. Long-term extinction was tested at 24 h after the re-exposure. The mice received a microinfusion of ALLN (2 μg/side) or VEH into the dorsal hippocampus at 10 min before (Fig. 3a and b) or immediately after (Fig. 3c and d) the re-exposure. Mice in the VEH and ALLN groups showed decreased freezing levels, over time with re-exposure (pre-re-exposure infusion: female, *F*(5,120) = 23.272, *p* < 0.05; male, *F*(5,95) = 27.700, *p* < 0.05; post-re-exposure infusion: female, *F*(5,130) = 60.161, *p* < 0.05; male, *F*(5,95) = 49.793, *p* < 0.05; Fig. 3a–d). Further, overall freezing levels did not significantly differ during re-exposure (pre-re-exposure infusion: female, *F*(1,24) = 0.391, *p* > 0.05; male, *F*(1,19) = 1.467, *p* > 0.05; post-re-exposure infusion: female, *F*(1,26) = 0.001, *p* > 0.05; male, *F*(1,19) = 0.514, *p* > 0.05; Fig. 3a–d). These results indicated that the VEH and ALLN groups displayed comparable within-session extinction. Importantly, observations from the pre-re-exposure group suggested that the inhibition of hippocampal calpain did not affect within-session extinction. A two-way ANOVA comparing the freezing scores during the last 5 min in the re-exposure session and test revealed no significant effect of drug and the drug × time (re-exposure vs. test) interaction (pre-re-exposure infusion: female, drug, *F*(1,48) = 0.684, *p* > 0.05; time, *F*(1,48) = 1.542, *p* > 0.05; interaction, *F*(1,48) = 0.039, *p* > 0.05; male, drug, *F*(1,38) = 0.711, *p* > 0.05; time, *F*(1,38) = 2.024, *p* > 0.05; interaction, *F*(1,38) = 0.008, *p* > 0.05; post-re-exposure infusion: female, drug, *F*(1,52) = 0.816, *p* > 0.05; time, *F*(1,52) = 5.344, *p* < 0.05; interaction, *F*(1,52) = 0.228, *p* > 0.05; male, drug, *F*(1,38) = 0.005, *p* > 0.05; time, *F*(1,38) = 6.364, *p* < 0.05; interaction, *F*(1,38) = 0.296, *p* > 0.05; Fig. 3a – d). Thus, the inhibition of hippocampal calpain had no effect on long-term extinction. Taken together, our results suggest that hippocampal calpain is not required for within-session and long-term extinction in both sexes.

### Calpain is required for c-fos induction when contextual fear memory is generated

It is possible that calpain contributes to the activation of gene expression that is required for the consolidation of contextual fear memory, since calpain activity is required for the modification of GluN2B, which occurs an upstream of activity-dependent gene expression in excitatory neurons [25, 30, 46, 47, 49, 58]. To assess this, we examined how inhibiting calpain in the hippocampus affected the induction of c-fos expression, which depends on neuronal activity [59–61].

**Fig. 4:**
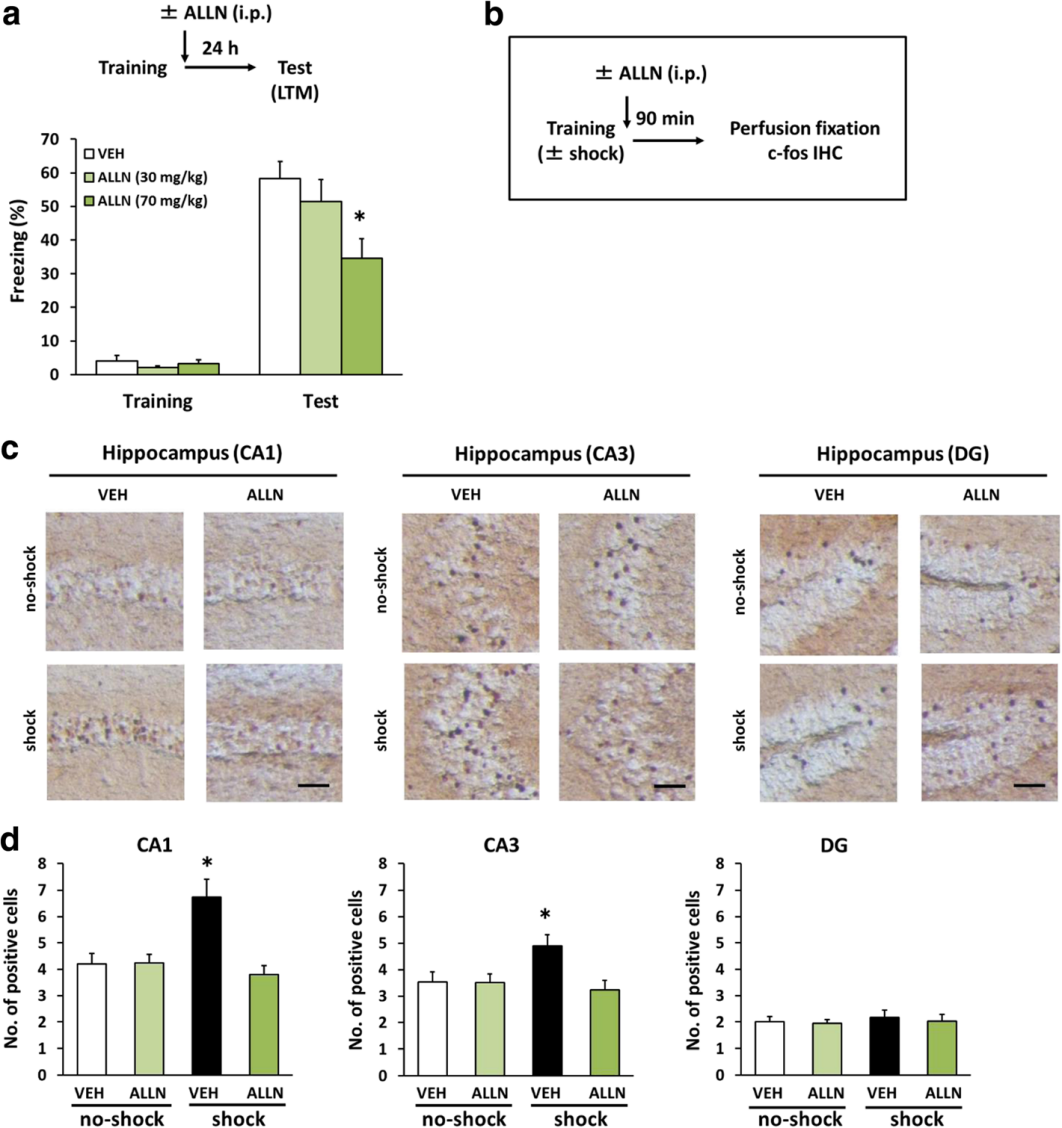
Inhibition of calpain blocks c-fos induction in the hippocampal CA1 and CA3 regions when memory is consolidated. **a** Effects of a systemic injection of a low- or high-dose of ALLN immediately after the training on LTM (VEH, *n* = 13; ALLN 30 mg/kg, *n* = 8; ALLN 70 mg/kg, *n* = 9). *p < 0.05, compared with the VEH group at the test. **b** Experimental design for IHC. **c** Representative immunohistochemical staining of c-fos-positive cells in the CA1, CA3, and DG regions of the indicated groups. Scale bar, 50 μm. **d** The number of c-fos-positive cells in the CA1, CA3, and DG regions of no-shock/VEH, no-shock/ALLN, shock/VEH, and shock/ALLN groups (*n* = 9 for each group). *p < 0.05, compared with the other groups. Error bars indicate SEM

We first examined the effects of a systemic injection of ALLN on the LTM of contextual fear at the behavioral level. We performed similar experiments to those outlined in Fig. 1, except the male mice were systemically injected with ALLN (low-dose, 30 mg/kg; high-dose, 70 mg/kg) or VEH immediately after the training. A one-way ANOVA revealed a significant drug effect (*F*(2,27) = 4.662, *p* < 0.05; Fig. 4a). Post-hoc Newman-Keuls analysis revealed that ALLN-treated mice froze significantly less, compared to VEH-treated mice, in a dose-dependent manner (low-dose, *p* > 0.05; high-dose, *p* < 0.05; Fig. 4a). Similar to Fig. 1, these observations indicated that the inhibition of calpain by ALLN inhibited the formation of contextual fear memory.

Next, we measured the number of c-fos-positive cells in the hippocampus (CA1, CA3, and dentate gyrus [DG]) of male mice at 90 min after the training using immunohistochemistry (IHC). Two groups were trained with a footshock (shock groups), while the remaining two groups did not receive a footshock (no-shock groups). These groups were systemically injected with ALLN (70 mg/kg) or VEH immediately after the training (the groups were as follows: shock/ALLN, shock/VEH, no-shock/ALLN, and no-shock/VEH groups; Fig. 4b). A two-way ANOVA revealed a significant shock × drug interaction in the CA1 and CA3 regions (CA1, shock, *F*(1,32) = 5.314, *p* < 0.05; drug, *F*(1,32) = 10.119, *p* < 0.05; interaction, *F*(1,32) = 10.862, *p* < 0.05; CA3, shock, *F*(1,32) = 2.208, *p* > 0.05; drug, *F*(1,32) = 5.23, *p* < 0.05; interaction, *F*(1,32) = 5.003, *p* < 0.05; Fig. 4c and d), but not in the DG region (shock, *F*(1,32) = 0.275, *p* > 0.05; drug, *F*(1,32) = 0.254, *p* > 0.05; interaction, *F*(1,32) = 0.03, *p* > 0.05; Fig. 4c and d). The shock/VEH group had significantly more c-fos-positive cells in the hippocampal CA1 and CA3 regions compared with the other groups, including the shock/ALLN group (*p* < 0.05; Fig. 4c and d). These results indicated that inhibition of calpain by ALLN blocked the c-fos induction in the hippocampus when memory is generated. This suggested that hippocampal calpain contributes to the activity–dependent gene expression when contextual fear memory is consolidated.

## Discussion

In the present study, we examined the roles of hippocampal calpain in the consolidation, reconsolidation, and extinction of contextual fear memory. Inhibiting hippocampal calpain by a local infusion of the calpain inhibitor ALLN blocked the formation of LTM, without affecting STM. Moreover, the inhibition of hippocampal calpain immediately after memory retrieval disrupted reactivated memory. Conversely, the inhibition of hippocampal calpain had no effect on long-term extinction. Therefore, these observations demonstrated that hippocampal calpain is required for the consolidation and reconsolidation, but not extinction, of contextual fear memory.

Importantly, previous studies showed that protein degradation is involved in the molecular processes necessary for synaptic plasticity and learning and memory [16–20]. Calpain is Ca^2+^-dependent cysteine protease involved in Ca^2+^ signaling pathway [21, 22]. Calpain specifically cleaves substrates in neurons, including synaptic proteins such as NMDA receptors subunits GluN2A and GluN2B, p35, calcineurin, alpha calcium/calmodulin-dependent protein kinase II (αCaMKII), spectrin, beta-catenin, and MAP2 [25, 26, 28–30, 32, 35, 36, 62–65]. Calpain is activated by NMDA receptor stimulation [30, 36, 66]. Activated calpain specifically cleaves the C-terminal of GluN2B, leading to degradation of NMDA receptors, which possibly modulates learning and synaptic plasticity [26, 30, 67, 68]. Activated calpain generates p25 by cleaving the N-terminal of the Cdk5 activator p35 [41]. Importantly, previous mouse genetic studies demonstrated that genetic deletion of p35 impaired hippocampus-dependent spatial learning and memory [39], whereas the transient or prolonged overexpression of p25 enhanced or impaired hippocampus dependent memory, respectively [42, 43]. Interestingly, Cdk5 facilitates the degradation of GluN2B by directly interacting with both it and calpain, suggesting crosstalk among calpain, NMDAR, and Cdk5 [40]. Taken together with our finding that hippocampal calpain is required for contextual fear memory consolidation and reconsolidation, it is possible that calpain in the hippocampus contributes to memory consolidation and reconsolidation through the functional modification of GluN2B and p35 by cleaving them.

Calpains, which are localized in spines [69, 70], have been suggested to mediate changes in the cytoskeletal structure and organization [42, 71] by cleaving substrate proteins [60, 61]. The genetic deletions of the calpain 1 / calpain 2 genes resulted in the decline in spine density and dendritic branching complexity in hippocampal CA1 pyramidal neurons, which further impaired the induction of LTP by theta burst stimulation in the CA1 area of the hippocampus [44, 72, 73]. Interestingly, recent studies have suggested distinct roles of calpain 1 and 2 in synaptic plasticity [45]; calpain 1 is required for the induction of LTP while calpain 2 is necessary for this maintenance. Moreover, deletions of calpain genes impaired hippocampus-dependent spatial learning in the Morris water maze [44]. In the current study, we extended these findings and demonstrated that hippocampal calpain is required for the consolidation and reconsolidation of contextual fear memory, but not for learning, short-term memory, and extinction memory. Further studies are required to understand the molecular mechanisms by which calpain contributes to the consolidation and reconsolidation by cleaving target substrates, and to compare and clarify roles of calpain 1 and 2 in these memory processes.

Additionally, we suggested that hippocampal calpain is not required for extinction of contextual fear memory, similarly with previous findings that long-term extinction does not require hippocampal gene expression. It is necessary to examine roles of calpain in the amygdala and mPFC in memory extinction since a previous study showed that extinction of contextual fear memory requires gene expression in these brain regions [48].

The activation of gene expression is necessary for the consolidation and reconsolidation of contextual fear memory [7, 15, 46–49, 58]. Interestingly, we showed that inhibiting calpain not only disrupted the consolidation of contextual fear memory, but also blocked the induction of c-fos expression that was observed following training. Calpains have been suggested to contribute to neuronal processes, including gene transcription and synaptic plasticity [32–34]. Therefore, it is possible that blocking the calpain inhibited the activation of gene expression, including the induction of c-fos expression, which is required for memory consolidation, since c-fos induction in hippocampal neurons is dependent on the activation of NMDA receptors [74–76]. Further studies are important to examine changes in cleavages of calpain targets such as beta-catenin following contextual fear conditioning to understand mechanisms for gene expression activation by calpain when memory is consolidated [65].

Sex differences had been observed in the molecular mechanisms that underlie the learning and memory process [50, 51]. However, the results of the current study did not demonstrate any sex differences in the role of hippocampal calpain in memory consolidation, reconsolidation, and extinction of contextual fear. This suggested that calpain is not involved in sex-specific molecular processes for memory performance.

Overall, the current study demonstrated that hippocampal calpain is necessary for both the consolidation and reconsolidation of contextual fear memory. Our findings suggested that calpain contributes to gene expression-dependent memory processes as a downstream regulator of the Ca^2+^-signal transduction pathway.

## Methods

### Mice

All experiments were conducted according to the *Guide for the Care and Use of Laboratory Animals* (Japan Neuroscience Society and Tokyo University of Agriculture). The Animal Care and Use Committee of Tokyo University of Agriculture (authorization #280020) approved all the animal experiments that were performed in this study. All surgical procedures were performed under Nembutal anesthesia, with every effort to minimize suffering. Male and female C57BL/6 N mice were obtained from Charles River (Yokohama, Japan). The mice were housed in cages of 5 or 6, maintained on a 12-h light/dark cycle, and allowed ad libitum access to food and water. The mice were at least 8 weeks of age at the start of the experiments, and all behavioral procedures were conducted during the light phase of the cycle. All experiments were conducted by researchers who were blinded to the treatment condition of the mice.

### Surgery for drug microinfusion

**Fig. 5:**
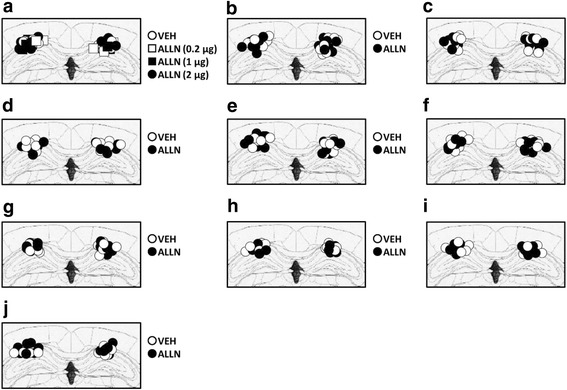
Cannula tip placements in the dorsal hippocampus. Cannula tip placements from mice infused with each drug shown in Fig. 1a (**a**), Fig. 1b (**b**), Fig. 1c (**c**), Fig. 1d (**d**), Fig. 2a (**e**), Fig. 2b (**f**), Fig. 3a (**g**), Fig. 3b (**h**), Fig. 3c (**i**), Fig. 3d (**j**). Schematic drawing of coronal sections from all micro-infused animals (dorsal hippocampus, 1.94 mm posterior to the bregma). Only mice with needle tips within the boundaries of the dorsal hippocampus were included in the data analysis

Surgeries were performed as described previously [56, 60, 61, 77–80]. Stainless-steel guide cannulae (22 gauge) were implanted into the dorsal hippocampus (−1.8 mm, ±1.8 mm, −1.9 mm), under Nembutal anesthesia, using standard stereotaxic procedures. The mice were allowed a recovery period of at least 1 week after surgery. Bilateral infusions into the dorsal hippocampus (0.5 μL/side) were made at a rate of 0.25 μL/min. The injection cannula was left in place for 2 min after infusion. Only mice with cannulation tips within the boundaries of the bilateral dorsal hippocampus were included in the data analysis. Cannulation tip placements are shown in Fig. 5.

### Drugs

The calpain inhibitor N-Acetyl-Leu-Leu-norleucinal (ALLN; 0.4, 2, or 4 μg/μL; Millipore, MA, USA) was dissolved in dimethyl sulfoxide with a final concentration that was less than 1% [81].

### Contextual fear conditioning task

The mice were handled for 5 consecutive days prior to the commencement of contextual fear conditioning. The mice were trained and tested in conditioning chambers (17.5 × 17.5 × 15 cm; O’HARA & Co., Ltd., Tokyo, Japan) that had a stainless-steel grid floor through which the footshock could be delivered [15, 48, 60, 61, 78, 82, 83]. Training consisted of placing the mice in the chamber and delivering an unsignaled footshock (2 s duration, 0.4 mA) 148 s later. Then, the mice were returned to their home cage at 30 s after the footshock (training).

For the experiments examining the effects of drug treatment on memory consolidation, the mice received a microinfusion of ALLN or vehicle (VEH) into the dorsal hippocampus immediately after training (see Fig. 1). At 2 h or 24 h after training, the mice were placed back in the training context for 5 min and freezing was assessed (test). For the experiments examining the effects of drug treatment on memory reconsolidation or extinction, the mice were trained and placed back in the training context 24 h later (re-exposure) for 3 min (reconsolidation) or 30 min (extinction). The mice received a microinfusion of ALLN or VEH into the dorsal hippocampus at 10 min before or immediately after re-exposure (as indicated in Figs. 2 and 3). At 24 h after the re-exposure, the mice were once again placed back in the training context for 5 min and freezing was assessed (test). Memory was assessed as the percentage of time spent freezing in the training context. Freezing behavior (defined as complete lack of movement, except for respiration) was measured automatically as described previously [84]. ALLN or VEH was systemically injected (an i.p. injection) immediately after training (see Fig. 4).

### Immunohistochemistry

Immunohistochemistry was performed as described previously [60, 61, 77–80, 85]. After anesthetization, all mice were perfused with 4% paraformaldehyde. Brains were then removed, fixed overnight, transferred to 30% sucrose, and stored at 4 °C. Coronal sections (30 μm) were cut using a cryostat. The sections were pretreated with 4% paraformaldehyde for 20 min and 3% H_2_O_2_ in methanol for 1 h, followed by incubation in blocking solution (phosphate-buffered saline [PBS] plus 1% goat serum albumin, 1 mg/mL bovine serum albumin, and 0.05% Triton X-100) for 3 h at 4 °C. Consecutive sections were incubated using a polyclonal rabbit primary antibody for anti-c-fos (1:5000; Millipore catalog #PC38, RRID: AB_2106755) in the blocking solution for 2 nights at 4 °C. Subsequently, the sections were washed with PBS and incubated for 4 h at room temperature with biotinylated goat anti-rabbit IgG (SAB-PO Kit; Nichirei Biosciences, Tokyo, Japan). Thereafter, the sections were incubated with streptavidin- biotin-peroxidase complex (SAB-PO Kit) for 1 h at room temperature. Immunoreactivity was detected using a DAB substrate kit (Nichirei Biosciences). Structures were anatomically defined according to the Paxinos and Franklin atlas [86]. Quantification of c-fos-positive cells in sections (100 × 100 μm) of the dorsal hippocampus (bregma between −1.46 and −1.82 mm) was performed using a computerized image analysis system (WinROOF version 5.6 software; Mitani Corporation, Fukui, Japan). Immunoreactive cells were counted bilaterally with a fixed sample window across at least 3 sections by an experimenter who was blinded to the treatment condition.

### Data analysis

One-way or two-way factorial analysis of variance (ANOVA) followed by post hoc Newman-Keuls comparisons were used to analyze the effects of drug, time, and shock. A two-way repeated ANOVA followed by a post hoc Bonferroni’s comparison was used to analyze the effects of drug and time. All values in the text and figure legends represent the mean ± standard error of the mean (SEM).

## Abbreviations

ALLN: N-Acetyl-Leu-Leu-norleucinal
Cdk5: Cyclin-dependent kinase 5
GluN2B: NMDA receptor 2B subunits
IHC: Immunohistochemistry
LTM: Long-term memory
LTP: Long-term potentiation
STM: Short-term memory
VEH: Vehicle; DG: Dentate gyrus
αCaMKII: Alpha calcium/calmodulin-dependent protein kinase II
